# Endoscopic Evaluation after Conventional Adenoid Curettage

**DOI:** 10.1055/s-0044-1779434

**Published:** 2024-02-16

**Authors:** Ahmed Abdelfattah Bayomy Nofal, Mohamed Abdelmohsen Alnemr, Ahmed Hassan Sweed, Alsayed Abdulmageed

**Affiliations:** 1 ENT Department, Reem Hospital, Abu Dhabi, United Arab Emirates; 2 Department of Otorhinolaryngology, Head and Neck Surgery, Faculty of Medicine, Zagazig University, Zagazig, Egypt

**Keywords:** adenoidectomy, endoscopy, nasopharynx, curettage

## Abstract

**Introduction** Adenoidectomy is one of the most common procedures performed by otolaryngologists. Traditional adenoid curettage is performed blindly, which can result in inadequate removal of the adenoid and injury to the surrounding structures.

**Objective** To perform transnasal endoscopic examinations to assess the nasopharynx after conventional curettage adenoidectomy.

**Methods** The present prospective study included 100 children with a mean age of 4.2 ± 3.07 years. It is composed of two steps: conventional curettage adenoidectomy by a resident trainee; and endoscopic evaluation of the nasopharynx through a 0° telescope to assess adenoidal remnants, injury to the surgical field or adjacent structures, and bleeding points.

**Results** Adenoid remnants were observed in 42% of the cases after conventional adenoid curettage in multiple locations, such as the roof of the nasopharynx over the choana (24%), the tubal tonsil (12%), the posterior pharyngeal wall (4%), and the posterior end of the nasal septum (2%). Injury to the surgical field and adjacent structures was observed in 46% of the cases (posterior pharyngeal wall: 23%; lateral pharyngeal wall: 11%; Passavant ridge: 10%; and the Eustachian tube orifice: 2%). Endoscopic bleeding was observed in 29% of the cases; 13% of the cases were from adenoid remnants, 10%, from the mucosa, and 6%, from the pharyngeal muscles. Bleeding was mild in 19% of the cases, moderate in 9%, and severe in 1%.

**Conclusion** Endoscopic evaluation of the nasopharynx following conventional adenoid curettage provides important data regarding adenoid remnants, injury to the surgical field or nearby structures, and bleeding points, which aids in the provision of optimal care and in the achievement of a better outcome.

## Introduction

Adenoidectomy is the surgical removal of adenoid tissue from the nasopharynx, and it is one of the most common and classic procedures performed by otolaryngologists. 1 Snoring, recurring upper airway infections, middle ear ventilation issues, and nasal blockage are all indications for an adenoidectomy, 2 3 which can be performed in isolation or combined with other ear, nose, and throat (ENT) surgical procedures, such as tonsillectomy or tympanostomy with insertion of a ventilation tube. 4

As with any other surgery, adenoidectomy may be associated with early and late postoperative morbidity and complications. Hemorrhage, which could be life-threatening, infection, aspiration, pulmonary edema, velopharyngeal incompetence, nasopharyngeal stenosis, atlantoaxial dislocation, mandibular condyle fracture, infection, Eustachian tube injury, and psychological stress are possible postadenoidectomy consequences. 5 6 The adenoid remnant in the nasopharynx is one of the important contributing elements, which only requires recurettage to reduce the initial postadenoidectomy bleeding. 7

Endoscopic instruments are now widely used, and there is an increasing number of successful surgical applications. Adenoidectomy methods with endoscopic assistance to remove additional adenoid tissue with Blakesley or Weil forceps via combined transoral and transnasal approaches after traditional curettage have been described and intend to provide direct visualization during the procedure, which makes it more effective. 8 9

Traditional adenoid curettage is frequently performed blindly, without direct visualization of the operative field, which can result in incomplete adenoid removal with some persistent adenoid remnants and injury to nearby structures or to the posterior pharyngeal wall. The aim of the present study is to perform endoscopic examinations to assess the nasopharynx and the surgical field after conventional curettage adenoidectomy to report the incidence and types of complications and to justify the importance of routine endoscopic assistance during the procedure.

## Methods

The present study included children who were adenoidectomy candidates at a university hospital, and it was authorized by the Institutional Review Board (ZU-IRB #10194/6–12–2022).

All children exhibited at least one of these symptoms: nasal obstruction, mouth breathing, snoring, trouble breathing while sleeping, and persistent earaches. An X-ray of the nasopharynx revealed an encroaching adenoid on the nasopharyngeal airway column. Children with congenital malformations, bleeding tendencies, or with a history of adenoidectomy were excluded.

All patients had a complete blood count (CBC), partial thromboplastin time, bleeding time, clotting time, and a preoperative (lateral view) nasopharynx X-ray of the soft tissue. None of the patients had upper respiratory tract infection at least two weeks prior to surgery, and they had not taken aspirin or other non-steroidal anti-inflammatory drugs in the previous ten days.

The study was composed of two steps:

The first step was conventional curettage adenoidectomy, as it is routinely performed by the resident: under general anesthesia with oral endotracheal tubes while the patient is in the supine position with the head extended with the use of a small shoulder sandbag. The mouth is opened with a Boyl Davis mouth gag to protract the tongue; then, digital palpation of the soft palate is performed to assess the submucous cleft, as well as of the nasopharynx, to assess the site and size of the adenoid and the degree of choanal occlusion. The hypertrophied adenoidal tissue is curetted several times with the a Beckman curette of appropriate size until the adenoid is eliminated. The nasopharynx and surgical field are digitally palpated again to examine the aperture of the posterior choana and to check for any palpable adenoid remnants.
  The nasopharynx is packed with saline-soaked ribbon gauze and left for at least 10 minutes. If it is combined with another surgery, the adenoidectomy is performed first, followed by the other procedure, and finally the nasopharyngeal pack is removed.
  The first step is the standard adenoidectomy procedure, which is performed by a resident physician under the supervision of specialists. If no bleeding occurs, the surgery is performed and the patient is woken and released, in a day-case procedure.
The second step was as follows: after the resident physician had completed the adenoidectomy surgery, the consultant physician used a the 0° telescope via the nose to check the nasopharynx and surgical field for the following:
▪ Presence of adenoidal tissue remnants in the roof of nasopharynx over the choana, in the lateral pharyngeal wall, the posterior pharyngeal wall, or the posterior wall of the nasal septum;
▪ Injury to the posterior or lateral pharyngeal walls; and
▪ Bleeding at the site of the adenoidectomy.

Following the reporting of the aforementioned points, any adenoidal remnants are either removed using the Blakesley forceps or ablated with bipolar cautery, and any injuries to the pharyngeal wall and hemorrhage are managed.

Bleeding after the adenoidectomy is classified as mild, moderate, and severe; mild bleeding is stopped through removal of the adenoid remnants, bipolar cautery of the bleeding point, or management of the pharyngeal wall injury; moderate bleeding does not stop completely with the aforementioned procedures and requires the application of nasopharyngeal packing for 15 minutes followed by re-examination of the nasopharynx with the 0° telescope; and severe bleeding is does not stop with the aforementioned procedures and requires posterior nasal packing for at least 24 hours.

## Results

The present study included 100 children, 42 boys and 58 girls, with a mean age of 4.2 ± 3.07 (range: 2 to 10) years. In total, 32 subjects underwent adenoidectomy in isolation, 38 were submitted to adenotonsillectomy, 13 underwent adenotonsillectomy with ventilation tubes, and 17 were submitted to adenotonsillectomy with ventilation tubes.

The mean duration of the traditional adenoid curettage procedure is of 16 ± 4.5 minutes, while that of the endoscopic evaluation of the nasopharynx following adenoidectomy with management of any remnants, injury, or bleeding is of 23 ± 7 minutes.

**Fig. 1:**
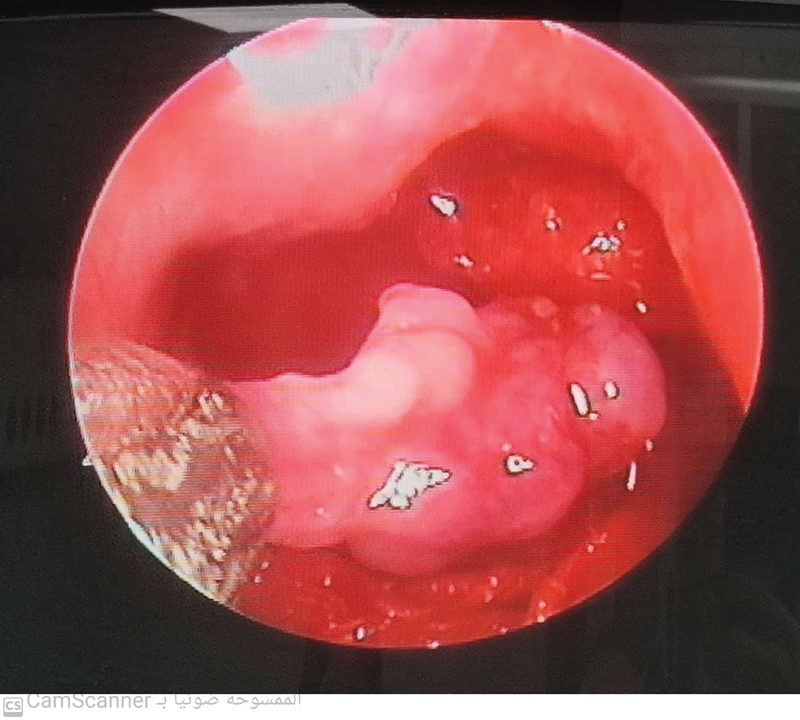
Large adenoid remnant in the choana, measuring 10 × 5 mm, observed through endoscopic evaluation.

The first assessment point is the adenoid remnants (any adenoid tissue measuring more than 4 × 4 mm at the site of the adenoid bed), which were observed in 42% of the cases through the endoscopic evaluation; in 24% of the cases, they were in the choanal region (either uni- or bilaterally); in 12% of the cases, they were in the area of the Eustachian tube orifice (in the lateral pharyngeal wall); in 4% of the cases, they were in the posterior pharyngeal wall; and in 2% of the cases, they in the posterior edge of the nasal septum. The size of the remnants ranged from 4 mm to 12 mm ( Fig. 1 ) ( Table 1 ).

**Table 1:** Endoscopic evaluation after conventional adenoid curettage

			A (%)	A&T (%)	A&T + V (%)	A + V (%)	Total (%)
Remnants	Site	Roof of the nasopharynx over the choana	9	7	4	4	24
		Tubal tonsil	3	2	4	3	12
		Posterior pharyngeal wall	1	2	−	1	4
		Posterior end of the nasal septum	1	−	1	−	2
Injury to nearby structures	Posterior pharyngeal wall		9	7	3	4	23
	Lateral pharyngeal wall		3	5	1	2	11
	Passavant ridge		3	5	2	−	10
	Eustachian tube orifice		1	−	−	1	2
	Posterior end of the nasal septum		−	−	−	−	0
Bleeding points	Site	Adenoid remnants	3	8	1	1	13
		Posterior pharyngeal wall mucosa	2	3	1	−	6
		Lateral pharyngeal wall mucosa	1	2	−	1	4
		Pharyngeal muscles	2	4	1	1	6
	Degree	Mild	6	11	1	1	19
		Moderate	3	5	1	−	9
		Severe	−	1	−	−	1

**Abbreviations:** A, adenoidectomy; A&T, adenoidectomy and tonsillectomy; A&T + V, adenoidectomy and tonsillectomy with insertion of a ventilation tube; A + V, adenoidectomy with insertion of a ventilation tube.

The second assessment point is the injury to the nearby structures, which was found in 46% of the cases: in 23%, it was to the posterior pharyngeal wall; in 11%, to the lateral pharyngeal wall in 11%; in 10%, to the Passavant ridge; and in 2% of the cases, to the Eustachian tube orifice. No injuries to the posterior part of the nasal septum were observed in any of the cases ( Table 1 ).

The third assessment point is bleeding, more specifically, the site and degree of bleeding ( Table 1 ). During endoscopic evaluation, bleeding was observed in 29% of the cases; in 13%, it was from the adenoid remnants; in 6%, from the mucosa of the posterior pharyngeal wall; in 4%, from the mucosa of the lateral pharyngeal wall; and, in 6%, from the pharyngeal muscles.

In 19% of the cases, the bleeding was mild, and it was controlled through removal of the adenoid remnants, bipolar cautery of the bleeding point, or management of the pharyngeal wall injury. In 9% of the cases, the bleeding was moderate, and it was controlled by applying nasopharyngeal packing for 10 to 15 minutes after the procedure. In 1% of the cases, the bleeding was severe, and it was controlled by posterior nasal packing for 24 hours.

## Discussion

Adenoidectomy can be performed using one of three techniques: standard curettage, electrocautery, or power-assisted curettage. Each strategy presents benefits as well as drawbacks. 6 10 11 12 13 14 15 16 17

With so many patients requesting adenoidectomy at our facility, two primary factors influence the treatment: the cost of the procedure and the expertise of the doctors. Traditional adenoid curettage presents the benefit of being the least expensive maneuver (no costs involving probes or blades) and is usually performed by a resident physician.

In their study, Lo and Rowe-Jones 10 faced a similar issue when performing adenoidectomy under direct vision, which is the limitation of scopes and the time required for decontamination in connection to the large number of cases.

In the present study, we hope to demonstrate that, while adenoid curettage appears to be a straightforward and quick technique, it has downsides that require greater attention and monitoring. In the context of the present study, we would like to justify the importance of routine endoscopic examination of the nasopharynx, which aids in the management of any remnants or bleeding immediately following the procedure; it also provides surgeons with a helpful notion of the postoperative surgical field and of the possibility of leaving remnants or injury to nearby structures, which will improve their skills and learning curve over time.

In the present study, after conventional adenoid curettage, in 42% of the cases the endoscopic assessment showed adenoid remnants with sizes ranging from 4 mm to 12 mm in multiple locations of the nasopharynx, such as the roof of the nasopharynx over the choana (24%), the tubal tonsil (12%), the posterior pharyngeal wall (4%), and the posterior end of the nasal septum (2%). These remnants may generate blockage or proliferation, resulting in symptoms that require subsequent revision surgery.

In the study by Regmi et al., 18 conventional curettage adenoidectomy failed to completely remove adenoid tissue from the superomedial choanae and anterior vault in all cases, and incomplete removal was also observed in other parts of the choanae (67%), the eustachian tube orifice (63%), the nasopharyngeal roof (62%), and the Rosenmüller fossa (61%). Moreover, in the study by Modi et al., traditional adenoidectomy showed a greater risk of persistence of adenoid tissue. 19

This shows that the conventional adenoid curettage with a Beckman curette is unable to reach every part of the nasopharynx to eliminate all adenoidal tissue, particularly when the adenoid extends to the nose through the choana or attaches to the posterior edge of the nasal septum, and that blind maneuvering was not helpful to the surgeon in assessing the field after removal.

Di Rienzo Businco et al. 6 reported the same observation that Beckman curettes cannot reach the most cranial area of the adenoid as well as the intranasal extension of the adenoid.

In the present study, tears and injuries to the surgical field and adjacent structures were observed in 46% of the cases in the postoperative endoscopic assessment, in regions such as the posterior pharyngeal wall (23%), the lateral pharyngeal wall (11%), the Passavant ridge (10%), the Eustachian tube orifice (2%). No injuries to the posterior end of the nasal septum were observed. These tears and injuries induce intra- and postoperative bleeding and increase postoperative pain, the risk of postoperative infection, the danger of nasopharyngeal stenosis, and the duration of the recovery from the procedure.

This is another example of how the traditional Beckman adenoid curette is traumatic not just to the surgical field but also to adjacent structures. We can explain the lack of injuries to the posterior end of the nasal septum by stating that the Beckman curette cannot reach this area; the traumatic injury produced by it was made worse by the blind performance of the maneuver, in which the surgeon relies solely on sensation and digital palpation.

In the present study, the rate of postoperative bleeding after conventional adenoid curettage with a Beckman curette was of 29%. Bleeding was either from the adenoid remnants (13%) or from injuries to the posterior and lateral pharyngeal walls (16%), and it required interventions either by removal of the adenoid remnants, bipolar cautery of the bleeding points, or management of the pharyngeal wall tear and injury, which resulted in control of the bleeding in 19% of the cases. In the remaining cases, it stopped with the addition of another step: nasopharyngeal packing for 15 minutes in 9% of the cases and for 24 hours in 1% of the cases.

Based on the three assessment points (adenoid remnants, injuries to the surgical field and surrounding structures, and bleeding points), we can conclude that blind adenoid curettage with a Beckman curette is not a satisfactory procedure to completely remove the adenoid tissue in a safe and effective manner, and many complications can occur and go undetected with this blind maneuver.

Although the endoscopic assessment after conventional adenoid curettage adds more time to the procedure, this difference is negligible considering the better surgical field, fewer complications and better patient outcomes.

In their study, Yaman et al. 20 concluded that transnasal endoscopic examination following conventional curettage adenoidectomy is an appropriate method to assess residual adenoid tissue that should be performed in every case.

As a result, we recommend performing adenoidectomy in a fashion that enables direct visualization throughout the process, or at the very least an endoscopic examination of the surgical field, to enable the early detection and management of any residual tissue, injuries, tears, or bleeding.

## Conclusion

Endoscopic assessment of the nasopharynx and surgical filed after traditional curettage adenoidectomy is simple, valuable, and affordable. It provides critically important data regarding any tissue remnants, injury to the surgical field or nearby structures, and bleeding points. This enables the provision of early and appropriate care regarding these issues, which also helps to enhance the learning curve of the surgeons, since these issues will be avoided in future procedures.
